# Physiological responses of sea urchin, *Arbacia punctulata*, exposed to temperature and lipopolysaccharides (LPS)

**DOI:** 10.1371/journal.pone.0344673

**Published:** 2026-03-10

**Authors:** Nahian Fyrose Fahim, Kusum Parajuli, Israt Mishu, Sinthia Kabir Mumu, Eaint Honey Aung Win, Ahmed Mustafa

**Affiliations:** 1 Department of Biological Sciences, Purdue University Fort Wayne, Fort Wayne, Indiana, United States of America; 2 Department of Biochemistry and Molecular Biology, The Pennsylvania State University, Pennsylvania, United States of America; Chang Gung University, TAIWAN

## Abstract

Sea urchins are interesting creatures that play important ecological roles in the sea and are popular for their culinary and medicinal uses, which belong to phylum of Echinodermata. However, rapid environmental changes create a significant impact on marine species, including sea urchins, causing them severe stress. To address this issue, scientists are attempting to cultivate sea urchins in aquaculture to aid both conservation and commercial efforts. In this study, we aimed to investigate the physiological effects of stressors on sea urchin *Arbacia punctulata*, using three different stress conditions: increased temperature as a physical stressor, inoculation of lipopolysaccharides (LPS) as a chemical stressor, and a combination of both (increased temperature and LPS). We collected coelomic fluid (CF) from all the experimental groups at day 1, day 3, day 7, and day 10 and observed significant variations in the numbers of total and differential coelomocytes, namely, phagocytic cells, vibratile cells, red spherule cells, and colorless spherule cells in different stress conditions compared to controlled conditions (p < 0.05). The immune cells of sea urchins, especially phagocytic cells and red spherule cells, actively responded with LPS (4 µg/ml of CF/day). Our study also found a significant amount of protein in sea urchin’s cell free coelomic fluid exposed to increased temperature stress (p < 0.05) compared to that of control group. Both physical and chemical stressors impacted the growth and reproduction of sea urchins for long time exposure to stressors. We also observed lower gonadosomatic index (GSI) in the group exposed combined stressors: LPS inoculation (4 µg/ml of CF/day) and increased temperature (1˚C/day) in comparison with the control group (p < 0.05) at day 10.

## 1. Introduction

Global warming and pollutants have considerable impacts on marine ecosystems. The Earth’s average temperature has risen by 1 °C since the industrial revolution, primarily due to increased greenhouse gas emissions [1]. Consequences such as sea-level rise, ocean acidification, and altered weather patterns are placing substantial physiological stress on marine organisms. Warmer waters lead to coral bleaching, and ocean acidification reduces the ability of many marine organisms to form shells or skeletons, lowering their survival [2]. Pollutants such as oil spills, plastics, and chemical waste further degrade marine ecosystems causing long-lasting ecological damage, harming wildlife, and allowing toxic chemicals to bioaccumulate through the food chain [3,4]. Marine echinoderms such as sea stars, sea urchins, and sea cucumbers are similarly affected by global warming and pollution [5]. Many echinoderms exhibit regenerative abilities, although the extent varies among classes and species [6]. Rising sea surface temperatures have been shown to alter echinoderm physiology and behavior, with heat stress leading to reduced growth, impaired reproduction, and increased mortality in vulnerable species [7].

Sea urchins are primarily herbivores that feed on algae, though many species show opportunistic omnivory by consuming detritus, small invertebrates, and organic particles when algae are scarce. They serve as prey for larger predators such as sea otters and seabirds [8]. Internally, sea urchins possess a water vascular system that powers tube-foot movement and supports respiration, excretion, and sensory functions [9]. Sea urchins have economic and research value, and their life cycle involves external fertilization, planktonic larval development, metamorphosis, and long adult lifespans sometimes exceeding 30 years [10].

Sea urchins exhibit notable physiological responses to environmental stressors. Elevated temperatures can alter metabolism, respiration, immunity, and reproduction, often increase metabolic demand and reduce energy reserves. Chemical pollutants likewise disrupt physiology and immune function. Increased pollution elevates exposure to pathogenic bacteria, including Gram-negative species whose lipopolysaccharides (LPS) can cause cellular damage, DNA degradation, and immune activation [11]. LPS acts as a potent pathogen-associated molecular pattern (PAMP), triggering pattern-recognition receptor (PRR)-mediated signaling pathways [12]. Because sea urchins rarely encounter stressors in isolation, rising seawater temperatures often coincide with elevated bacterial loads and organic pollution. Our objective was to determine whether thermal stress amplifies LPS-induced immune challenges and whether combined exposure produces greater physiological disruption than stressor alone; an important consideration for understanding sea urchin resilience under climate change and for aquaculture management. In this experiment, we investigated the physiological and immunological responses of the common sea urchin *Arbacia punctulata* under stress exposure. *A. punctulata* is distributed throughout the western Atlantic from Newfoundland to the Gulf of Mexico and inhabits shallow rocky shores, seagrass beds, and sandy substrates. It is also commercially harvested for its roe, and its biological characteristics make it a valuable model for marine research [13]. We measured four key physiological biomarkers widely used to assess stress and immune disruption in echinoderms: coelomocyte abundance, coelomic-fluid (CF) protein concentration, ammonia excretion, and gonadosomatic index (GSI). Coelomocytes are the primary innate immune cells in sea urchins and rapidly change in number and composition in response to temperature stress, heavy metals, and pathogen-associated molecules such as LPS [11,14,15]. CF protein concentration reflects the presence of immune effector molecules including complement-like proteins, antimicrobial peptides, and other stress-responsive factors and typically increases under thermal or chemical stress due to heightened metabolic and immune activity [16]. Ammonia excretion serves as an indicator of metabolic rate and excretory load, both strongly influenced by temperature. Elevated ammonia excretion has been documented in sea urchins under environmental stress and is associated with increased metabolic demand and nitrogenous waste production [17,18]. GSI is a standard measure of reproductive condition and is among the earliest physiological traits to decline under chronic environmental stress, including elevated temperature, food limitation, and chemical or microbial exposure [19,20].

## 2. Materials and Methods

### 2.1. Animal acquisition and maintenance

Thirty-six live adult specimens of *Arbacia punctulata* were obtained from the Gulf Specimen Marine Laboratory in Florida, with a test diameter of 38 ± 2 mm. Upon arrival, the sea urchins were acclimated to circulating seawater with a salinity of 29 ± 1 ppt, temperature of 20 ± 1˚C, and pH of 8.0 ± 0.1 for 24 hours. Each tank had a 10-gallon saltwater reservoir and an AquaClear CycleGuard Power Filter for waste filtration at a rate of 12 L/hour. Thermostat aquarium heaters were used to regulate the water temperature, and floating aquarium thermometers were used to monitor the temperature in each tank. The pH and ammonia levels were checked regularly using an API Saltwater Aquarium Master Test Kit. Salinity of the water was measured with a refractometer. The sea urchins were fed daily with sea kelp from the supplier.

### 2.2. Exposure of stressors with LPS and Increased temperature

After the acclimation period, the sea urchins were kept in four experimental tanks with three biological replicates in each. During initial screening we have observed average coelomic fluid volume of sea urchin was around ~5 ml. Besides the control, three experimental groups were exposed to stressors: increased temperature (1°C per day till day 7), inoculation of 4 µg/ml of coelomic fluid /day lipopolysaccharides (LPS), and a combination of LPS and increased temperature. The lipopolysaccharide (LPS) preparation (Escherichia coli O26:B6; Sigma-Aldrich, Cat. No. L2654) used in this study is purified by phenol extraction and is reported by the manufacturer to be ≥ 97% pure. We acknowledge that the remaining ~3% may contain trace bacterial components such as peptidoglycan (PGN) or lipoproteins, which could contribute to immune activation. LPS was suspended in sterilized artificial sea water with an initial concentration 100 µg/ml and 0.2 ml was injected into the coelomic cavity through the peristomal membrane to each sea urchin in the LPS stressed group and combined stressed with a final concentration of 4 µg/ml of CF every day at 24 hours’ time interval. The 4 µg/ml of CF /day LPS dose was chosen based on concentrations previously shown to activate coelomocytes without causing rapid mortality in sea urchins. Studies in *Paracentrotus lividus* and *Strongylocentrotus intermedius* have used similar ranges (1–2 µg/ml of CF) to reliably induce measurable immune and physiological responses [21,22]. Our preliminary trials indicated that with increase concentration 4 µg/ml of CF /day was also to produce consistent immune activation (increased coelomocytes, RSCs, and phagocytic cells) while maintaining survivorship throughout the 10-day experiment.

### 2.3. Collection of coelomic fluid and sample preparation

The coelomic fluid (CF) was collected and handled according to the method described by Smith *et al.* (2018) with slight modifications [12]. CF of *Arbacia punctulata* was harvested by bleeding through the peristomal membrane using a 26-gauge hypodermic needle attached to a 1 ml sterile syringe following a standard protocol [23] with slight modifications. Every alternate day, 200 µl of coelomic fluid was withdrawn from each sea urchin, which included coelomocytes in the coelomic fluid (not in the peripheral tissue). The average volume was estimated to be approximately 5% of the total volume of the coelomic fluid in adult sea urchins.

### 2.4. Total and differential cell count

We counted four different types of immune cells of sea urchins: phagocytic cells, red spherule cells, colorless spherule cells, and vibratile cells suspended in the coelomic fluid. To estimate the number of cells, present in the coelomic fluid of sea urchins, we utilized the cell count method as described by previously stated protocol [24]. This involved obtaining 10µl of coelomic fluid from each sea urchin and placing it on a hemocytometer, a specialized counting chamber that allows for accurate cell counting under a microscope. Different types of cells in the top left and right (16 squares) of the hemocytometer were counted separately under a microscope (400x) and then averaged. To obtain cells per milliliter, we utilized the following equation:


$$\text{Cells}/\text{ml}=\text{ Average cell counts of 16 squares }\times \;(\text{10}{)}^{4}$$

### 2.5. Total protein

The measurement of total coelomic fluid protein was conducted using a Protein Refractometer (VEEGEE Scientific Inc. Kirkland, WA). Protein concentration measurements were performed using three independent biological replicates per treatment group. The refractometer resolution was ± 0.1 g/100 ml; SEM values of 0.00 reflect rounding to instrument precision limits. Coelomic fluid was put into a glass capillary tube, which was then sealed on one side with a Crito-cap. To separate coelomocytes from coelomic fluid, the capillary tubes were spun in a micro-hematocrit centrifuge at 10,000 rpm for 10 minutes. After that, two to three drops of coelomic fluid were placed on the surface of the prism of the calibrated refractometer. Afterward, the coelomic protein (g/100 ml) was read by holding the refractometer under the light.

### 2.6. Gonad somatic index

On day 10, terminal day of sampling, the sea urchin’s total weight is measured using a digital scale. The gonads are then carefully dissected out and weighed separately. The GSI is calculated as the ratio of gonad weight to total weight multiplied by 100. This method is based on the protocol described by [19]. The GSI is an important parameter for assessing the reproductive status, and growth of sea urchins and can provide valuable information for aquaculture and fisheries management [20]


Gonadosomaticindex= (Gonadweight/Totalweight) ×100

### 2.7. Statistical analysis

The data presented in this study are reported as mean ± standard mean error (SEM) with a sample size of three. Statistical analysis was conducted using Sigma Plot version 14.5. One-way analysis of variance (ANOVA) followed by the Bonferroni test was used to determine the differences among means. The significance level was set at p < 0.05 to indicate the major difference among means.

## 3. Results

**Fig 1:**
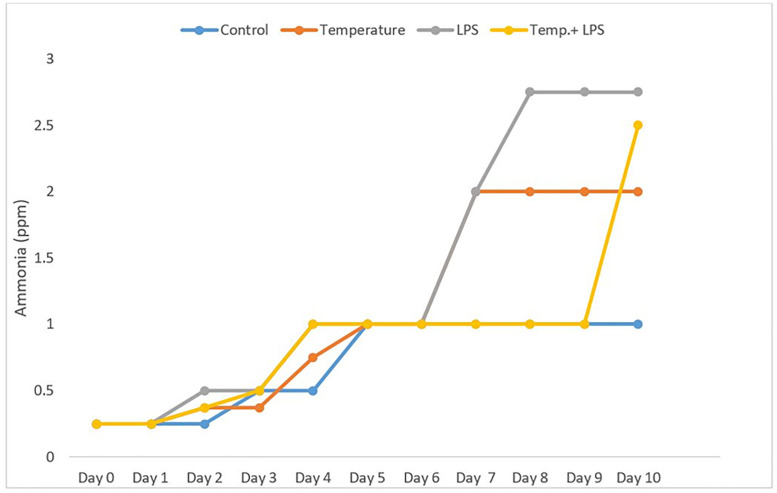
Level of ammonia (NH_3_/NH_4_^+^) (ppm) in the water of all four experimental tanks after exposure to the stressors (increased temperature and inoculation of LPS) in different days.

We estimated the water ammonia concentration every 24-hour interval to observe the stress responses in compared to control. Ammonia is one of the important factors to consider for the growth, reproduction, and survival of sea urchins. Optimal ammonia concentrations in marine aquaculture systems are typically maintained below 0.25 ppm to avoid sublethal toxicity in echinoderms [18]. Fig 1 shows at day 0 (acclamation period) and at day 1 ammonia concentration for all four experimental groups (control, 1°C Increased temperature, inoculation of 4 µg/ml LPS and combination of 1° increased temperature, and 4 µg/ml LPS) were less than or equal 0.25 ppm. On day 3 we noticed an increasing trend of ammonia. But on day 4, the amount of ammonia was 1 ppm in LPS stressed group (4 µg/ml/day) and the combined stressed group (4 µg/ml/day LPS and 4°c increased temperature). On day 7 ammonia concentration was recorded 2 ppm in LPS stressed group and temperature-stressed group (7°C). On days 8, 9, and 10 we recorded the highest ammonia concentration (~2.75 ppm) in LPS stressed group and the second highest ammonia concentration (~2.00 ppm) in temperature stressed group. Surprisingly, the level of ammonia also spiked from 1 ppm to 2.5 ppm for the combined stressed group. These results clearly demonstrate that the level of ammonia increased with the exposure of stressors compared to control.

**Fig 2:**
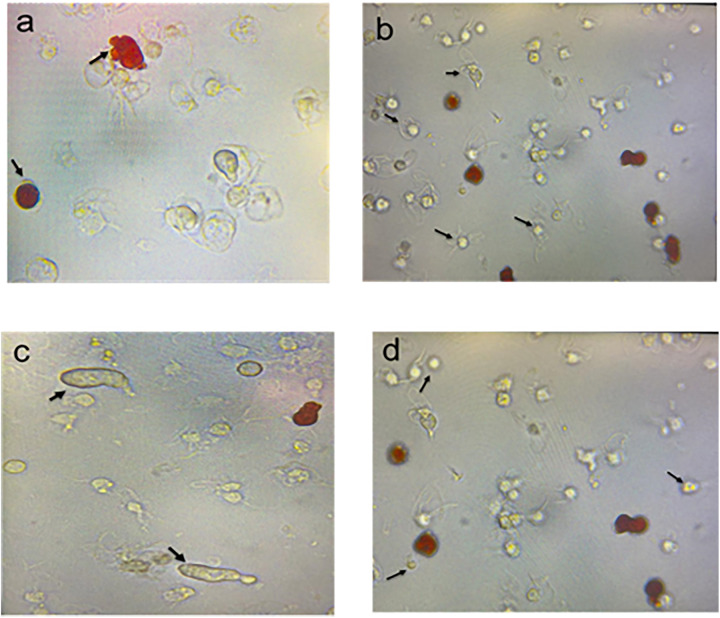
Four types of immune cells in sea urchin *Arbacia punctulata* under a compound microscope (400X) suspended in coelomic fluid; a) red spherule cells, b) phagocytic cells c) colorless spherule cells d) vibratile cells.

Sea urchins have a complex immune system composed of several specialized immune cells. Colorless spherule cells produce antimicrobial peptides, while red spherule cells produce reactive oxygen species to eliminate pathogens (Fig 2a and 2c) [7]. Red spherule cells are named for the reddish hue of their granules, though red spherule cells do not contain hemoglobin; instead, their granules are filled with echinochrome A, a naphthoquinone pigment that functions in antimicrobial defense and oxidative stress regulation [7,25,26]. Colorless spherule cells, on the other hand, have clear granules and play a critical role in immune functioning [12]. Phagocytic cells play an important role in phagocytosis, the process of engulfing and destroying pathogens. Phagocytic cells are generally small and round, with a large nucleus and granular cytoplasm (Fig 2b). Vibratile cells play a role in the overall defense against infections and are involved in the response to invading pathogens (Fig 2d) [26].

**Fig 3:**
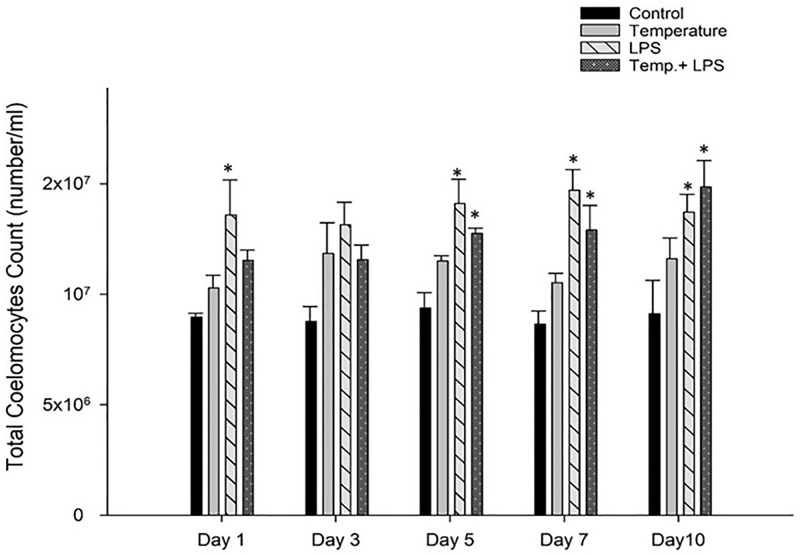
Total coelomocytes count (mean± SEM) of *Arbacia punctulata* after exposure to increased temperature (1°C per day till day 7), LPS inoculation (4 µg/ml/day), and LPS plus increased temperature as stressors. Differences were statistically significant at p < 0.05. The asterisk (*) shows significant differences compared to the control group within the specific sampling period.

Fig 3 shows the LPS stressed group had higher coelomocytes counts on each sampling day compared to control group (p < 0.05). At days 5, 7, and 10 the number of coelomocytes were also significantly higher in the combined stressed group (at 5°C, 6°C and 7°C temperature increase with 4 µg/ml/day LPS inoculation) in comparison to control (p < 0.05). Overall, LPS caused more stress in *Arbacia punctulata* than increase in temperature.

**Fig 4:**
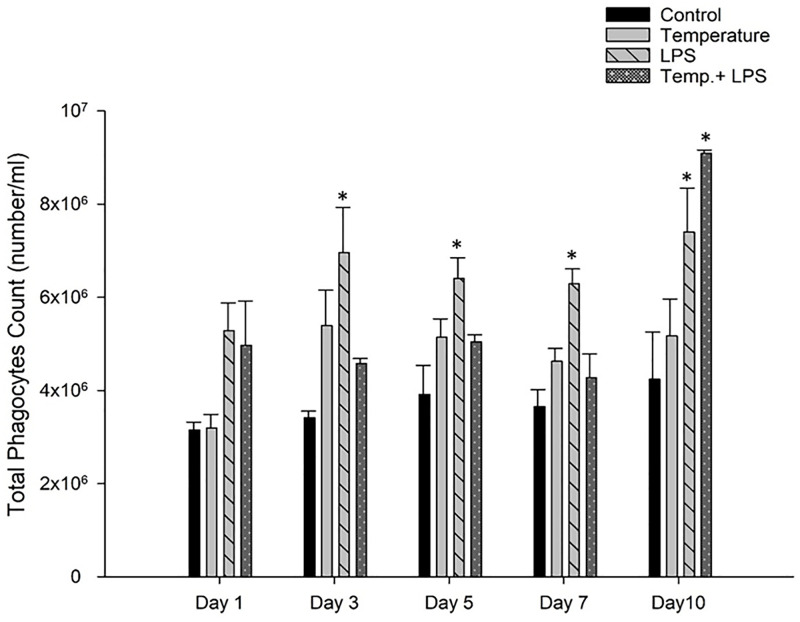
Phagocytic cells (mean± SEM) of *Arbacia punctulata* after exposure of increased temperature (1°C per day till day 7), LPS inoculation (4 µg/ml/day), and LPS plus increased temperature as stressors. Differences were statistically significant at p < 0.05. The asterisk (*) shows significant differences compared to the control group within the specific sampling period.

Phagocytic cells of sea urchins are involved with the cellular defense mechanism as they eliminate antigens, damaged cells, and microbial pathogens [12]. Fig 4 shows higher phagocytic cells count of *Arbacia punctulata* in LPS stressed group from day 3 to day 10 (terminal sampling day) than control (p < 0.05). Though from day 1 to day 7 the number of phagocytic cells was not significantly higher in the combined stressed group in comparison with control group, but it was significantly higher at day 10 (p < 0.05).

**Fig 5:**
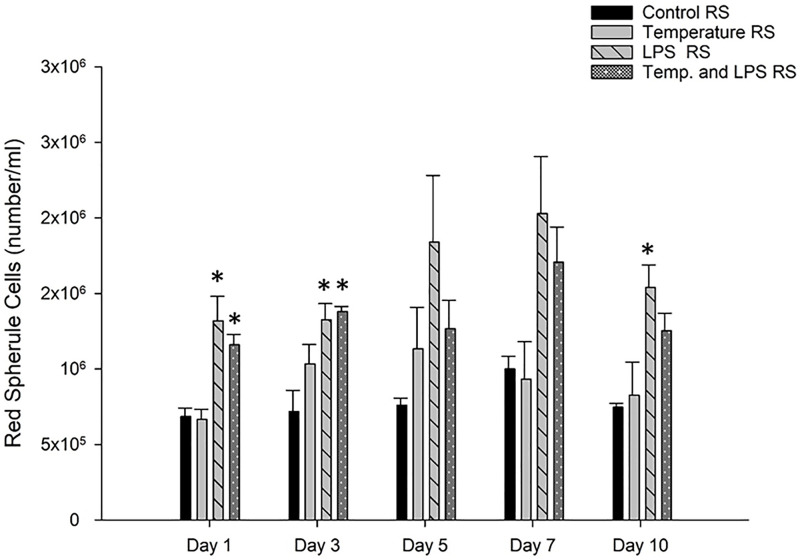
Red spherule cells (number/ml) count (mean± SEM) of *Arbacia punctulata* after exposure to increased temperature (1°C per day till day 7), LPS inoculation (4 µg/ml/day), and LPS plus increased temperature as stressors. Differences were statistically significant at p < 0.05. The asterisk (*) shows significant differences compared to the control group within the specific sampling period.

Red Spherule Cells (RSCs) are also involved in the immune response of sea urchins against pathogens, and vulnerable environments. In Fig 5, day 1 (1°C increased temperature and 4 µg/ml LPS) and day 3 (3°C increased temperature and 4 µg/ml/day LPS) data representing a significantly higher number (p < 0.05) of RSCs in LPS stressed group and combined stressed group (LPS and increased temperature) than the control group. On day 5 and day 10, we observed a greater number of RSCs in LPS stressed group and combined stressed group though results were not statistically significant (p > 0.05). On day 10 again we found a significant number of red spherule cells in LPS stressed group.

**Fig 6:**
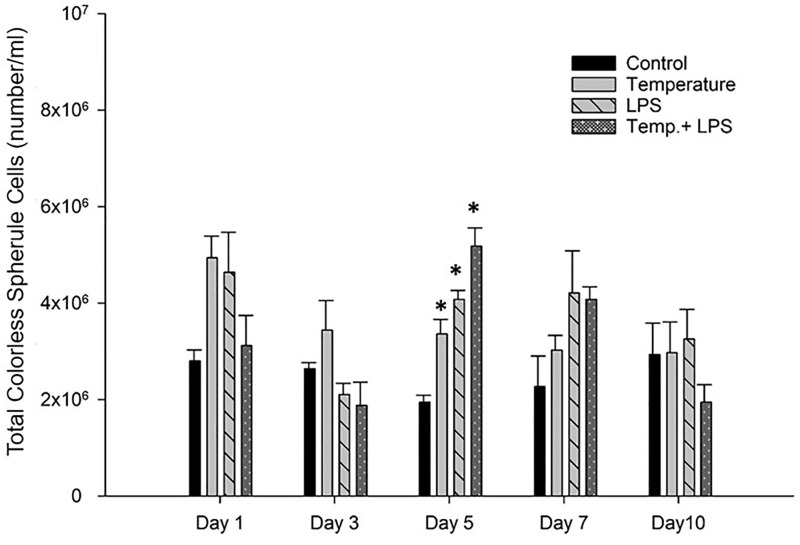
Colorless spherule cells (number/ml) count (mean± SEM) of *Arbacia punctulata* after exposure to increased temperature (1°C per day till day 7), LPS inoculation (4 µg/ml/day), and LPS plus increased temperature as stressors. Differences were statistically significant at p < 0.05. The asterisk (*) shows significant differences compared to the control group within the specific sampling periods.

Colorless spherule cells produce innate immune responses [12]. Fig 6 shows a significantly higher (p < 0.05) number of colorless spherule cells count at day 5 increased temperature (5°C) stressed, LPS stressed (4 µg/ml/day) and combined stressed group than those of the control group. Though a similar trend was observed on day 1 and day 7 results were statistically insignificant (p > 0.05).

**Fig 7:**
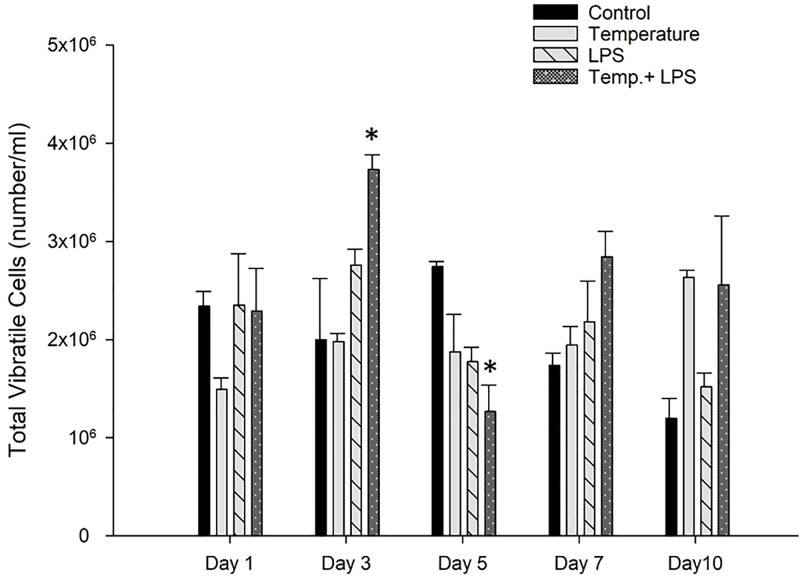
Total vibratile cells count (mean± SEM) of *Arbacia punctulata* after exposure of increased temperature (1°C per day till day 7), LPS inoculation (4 µg/ml/day), and LPS plus increased temperature as stressors. Differences were statistically significant at p < 0.05. The asterisk (*) shows significant differences compared to the control group within the specific sampling period.

Fig 7 shows the highest amount of vibratile cell count (VCCs) at day 3 in the combined stressed group (3°C increase of temperature and 4 µg/ml/day LPS) in comparison with the control group. Interestingly the lowest amount of vibratile cells was also observed on day 5 in the same stress group.

Table 1 shows combined stressed group (3°C increased temperature and 4 µg/ml/day LPS) had significantly different protein concentrations (1.80 ± 0.00 g/100 ml) in comparison to the control group on day 3 whereas at day 5 °C increased temperature stressed group had higher protein concentration than the control group. At days 7 and 10, again combined stressed group (7°C increased temperature and 4 µg/ml/day LPS) showed higher protein concentration (2.70 ± 0.05 g/100 ml and 2.60 ± 0.00 g/100 ml respectively) than the control though the highest protein concentration (2.80 ± 0.00g/100 ml) was observed in 7°C increased temperature stressed group at day 10.

**Table 1:** Coelomic protein (g/100 ml) of *Arbacia punctulata* after exposure to increased temperature, lipopolysaccharides (LPS), and LPS plus increased temperature as stressors in different sampling days. The concentrations are illustrated in mean ± SEM. The asterisk (*) shows significant differences in concentrations of coelomocyte protein in comparison to the control (p < 0.05).

Sampling Days	Control Group (g/100 ml)	Increased Temperature Group (g/100 ml)	LPS Group (g/100 ml)	LPS + Increased Temperature Group (g/100 ml)
Day 1	1.87 ± 0.06	1.87 ± 0.06	1.73 ± 0.06	1.80 ± 0.00
Day 3	2.07 ± 0.06	1.97 ± 0.03	1.9 ± 0.05	1.80 ± 0.00*
Day 5	2.10 ± 0.00	2.40 ± 0.10*	2.10 ± 0.00	2.37 ± 0.06
Day 7	2.20 ± 0.00	2.40 ± 0.05	2.30 ± 0.00	2.70 ± 0.05*
Day 10	2.10 ± 0.00	2.80 ± 0.00*	2.10 ± 0.00	2.60 ± 0.00*

**Fig 8:**
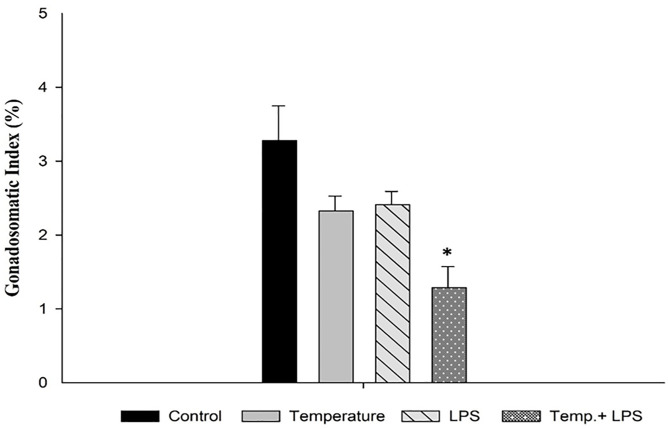
Gonadosomatic Index (%) of *Arbacia punctulata* after exposure of increased temperature (7°C for 10 days), lipopolysaccharides (LPS) (4 µg/ml/day), and LPS plus increased temperature (7°C for 10 days and 4 µg/ml/day LPS) as stressors. The asterisk (*) shows significant differences compared to the control group within a specific sampling period (p < 0.05).

The gonadosomatic index (%) is an important parameter to monitor reproductive activity and assess the health of sea urchins. Fig 8 shows the combined stressed group (7°C increased temperature then optimal condition and continuous exposure of 4 µg/ml/day LPS exposure for 10 days) of *Arbacia punctulata* had lowest (1.28) gonadosomatic index (p < 0.05) in comparison to the control. GSI of temperatures stressed group (2.32) and LPS stressed group were also lower than control group though the results were not statistically significant (p > 0.05).

## 4. Discussion

This study showed that sea urchin (*Arbacia punctulata)* responded with both physical and chemical stressors by activating their immune system and metabolic functioning. We monitored ammonia concentration as one of the parameters to understand the echinoderms’ health [21]. Our study showed both LPS (4 µg/ml/day) and temperature stress (1°C increase per day) have impacted the ammonia secretion rate of sea urchins, which is an important physiological response that helps to maintain acid-base balance and regulates internal pH. We observed higher concentration of ammonia in LPS stressed group and the combined stress group. A study by [27] found that exposure to stressors lead to a significant increase in ammonia secretion rates in the sea urchin *Strongylocentrotus intermedius.* Similarly, studies by [17] found that exposure to elevated temperature and low pH leads to an increase in ammonia excretion rates in the sea urchin *Paracentrotus lividus*. Research suggests that increased ammonia excretion in sea urchins is a physiological response to elevated metabolic demands under stress [18]. Our findings support this pattern: exposure to stressors increased metabolic activity to activate immune defenses, which resulted in higher ammonia levels in the stressed groups compared to the control (Fig 1). Because our goal was to examine the acute immune response to LPS, we used a relatively high LPS concentration for 10 days. Previous studies have shown that a temperature increase of 2–4 °C can significantly elevate ammonia excretion rates in sea urchins [17,28], although the exact exposure duration was not specified. Another study found that sea urchins exposed to 10 µg/mL LPS for 6 hours exhibited a significant rise in ammonia excretion [29].

Coelomocytes are the primary immune cells of sea urchins and are highly responsive to environmental stressors such as pollution, temperature changes, and pathogens [12]. Stress exposure, including heavy metals and bacterial infections, has been shown to increase total coelomocyte counts [21], highlighting their central role in echinoderm immunity [12]. Their major function is phagocytosis, through which they ingest and degrade microbes and damaged cells to prevent infection [29,12]. Sea urchin coelomocytes include four main types (Fig 2): red spherule cells, which respond strongly to hypoxia, heat, and heavy metal exposure [25]; phagocytic cells, which engulf bacteria and show enhanced activity after antigen exposure [30]; colorless spherule cells, which regulate reactive oxygen species [26]; and vibratile cells, which circulate nutrients, remove waste, and help maintain ion water balance [13,31]. Together, these cell types coordinate the innate immune response and reflect the sea urchin’s physiological condition under stress. A study Strongylocentrotus *purpuratus* showed exposure to LPS resulted in a significant increase in the total coelomocyte counts [14] which supported our findings (Fig 3). We observed a significant increase in total coelomocyte count (p < 0.05) in LPS stressed group and combined stressed group (LPS and increased temperature) compared to those of the control group. This increase in coelomocyte count is likely due to the activation of immune responses, including the recruitment of immune cells from other tissues. However, chronic exposure to LPS can also have a negative impact on the total coelomocyte count in sea urchins. In the sea urchin *Paracentrotus lividus,* prolonged exposure to LPS resulted in a decrease in the total coelomocyte count [21]. This decrease in coelomocyte count is likely due to the negative impact of chronic exposure to LPS on immune function and coelomocyte viability.

Changes in temperature can also impact coelomocyte counts in sea urchins. In the sea urchin *Paracentrotus lividus*, changes in temperature led to alterations in the count and activity of coelomocytes [32] but we did not observe any significant increase or decrease of coelomocyte count for temperature stressed group in *Arbacia punctulata* rather the combination of both stressors on day 10 showed highest coelomocyte count (Fig 3).

Exposure to lipopolysaccharides (LPS) can also have an impact on the count of specific types of coelomocytes, such as red spherule cells, colorless spherule cells, and phagocytic cells in sea urchins. Studies have shown that exposure to LPS can increase the count of red spherule cells in sea urchins [22]. For instance, in the sea urchin, *Paracentrotus lividus,* LPS exposure increased the count of red spherule cells in a concentration-dependent manner [26]. Similarly, in the sea urchin, *Strongylocentrotus intermedius*, exposure to LPS resulted in an increase in the count of red spherule cells. Studies have shown that the red spherule cell (RSC) count can serve as an indicator of acute stress response in sea urchins [12]. In our study, we observed a significant increase (p < 0.05) of red spherule cells in the LPS stressed group and the combined stressed group on day one and day three compared to the control group. A similar trend was observed on day 5 and day 7. As temperature stressed group did not show any significant increase or decrease in RSCs it can assume that LPS caused the highest stress in both groups (LPS stressed and combined stressed).

A study found that RSCs significantly increased in response to acute stressors such as predation and low oxygen levels in the sea urchin [33]. Similarly, a study found that RSCs increased in response to acute stressors such as mechanical stress, heat shock, and exposure to copper ions in the sea urchin [13]. These findings suggest that RSC count can be a useful tool for assessing the stress response in sea urchins. However, it is essential to note that the specific stress response may vary depending on the species of sea urchin and the type of stressor. In contrast, chronic exposure to LPS can have a negative impact on the count of colorless spherule cells and phagocytic cells in sea urchins. In *Arbacia lixula* and *Lytechinus variegatus*, *E. coli* challenge caused rapid and dynamic shifts in coelomocyte populations, characterized by a significant rise in phagocytes peaking at 48–72 hours [15]. Our findings indicate that although LPS alone had a stronger impact on phagocytic cell counts than temperature, the combined exposure to LPS and elevated temperature produced the highest phagocyte levels on day 10 because chronic warming amplified metabolic activity, increased immune sensitivity to LPS, and generated a cumulative synergistic stress response. The stressed groups showed significant differences in vibratile cell counts because vibratile cells are rapidly depleted or redistributed during immune activation and physiological stress. Our findings did not show any consistent vibratile cells counts among stressed groups (Fig 7), but it was obvious that combined stressors (increased temperatures and LPS) impacted the immune system and changes the balance in vibratile cells counts. Studies showed LPS triggers clotting and immune responses that reduce vibratile cell abundance, while increased temperature disrupts circulatory and osmoregulatory balance [21,32]. Cell-free coelomic fluid (CF) is the fluid that fills the body cavity (coelom) of sea urchins. CF plays vital roles in many physiological processes, including transport of nutrients and waste products, maintenance of water balance, and immune defense.CF contains a complex mixture of molecules, including proteins, lipids, carbohydrates, nucleic acids, and ions [16]. The proteins that remain in the coelomic fluid have been shown to play a role in the response of sea urchins to stressors such as LPS and changes in temperature. Studies have investigated the changes in CF protein expression in response to these stressors and have found that CF proteins may have a regulatory role in the immune response of sea urchins. According to research, in the sea urchin *Strongylocentrotus intermedius* [34], exposure to LPS resulted in changes in the expression of CF proteins involved in immune response pathways, such as the complement system (SpC3), NF-κB–related signaling and accumulation Sp185/333 proteins. Similarly, changes in temperature have been shown to alter the expression of CF proteins in sea urchins. High temperatures can cause thermal stress and activate the immune system, leading to the production of heat shock proteins (HSPs) and other immune-related proteins [35,36]. Our findings showed higher temperatures had a significant role in increasing the coelomic protein concentration (Table 1). We did not observe any significant increase of coelomic protein in LPS stressed group but increased temperature (3°C to 7°C) and combined stressed group showed a substantial increase of coelomic protein in *Arbacia punctulata.* We acknowledge that refractometry measures total dissolved solids rather than protein exclusively, and values may be influenced by salts or metabolic byproducts such as ammonia. Although refractometry is widely used in aquaculture for rapid CF protein estimation, future studies should employ Bradford or BCA assays for greater specificity and to distinguish true protein increases from osmotic or nitrogenous changes.

As stressors, both increased temperature and LPS inoculation can have a significant impact on the gonadosomatic index (GSI) of sea urchins, including *Arbacia punctulata,* but the specific effects may depend on the duration and intensity of exposure, as well as other factors such as the species and life stage of sea urchins. Some studies have suggested that exposure to LPS can have a greater effect on the GSI of sea urchins than increased temperature alone. For example, one study found that exposure to LPS caused a more significant reduction in GSI than a temperature increase [37]. According to another study, to achieve optimal production of sea urchin gonads temperature regulation is mandatory [38]. They observed maximum gonadal and somatic growth at 22°C temperature. Our findings demonstrated that combined exposure of LPS (4ug/ml/day) for 10 days and a 7°C increase in temperature caused a greater decrease (p < 0.05) of GSI in sea urchin *Arbacia punctulata* in comparison to control group. We also observed a decrease of gonaosomatic index the LPS stressed group and temperature (7°C increase) in comparison to stressed group. We have observed discoloration in the gonads in comparison to control. Our hypothesis is that increasing temperature and continuous exposure of higher dose of LPS is responsible for this, but still further experiment will describe the detailed.

Although LPS is widely used as a model PAMP in echinoderm immunology, it is important to note that the molecular mechanisms of LPS recognition in echinoderms remain incompletely understood. Recent work suggests that lipopolysaccharide detection by the innate immune system may not be a universally conserved defense strategy across metazoans [39]. Furthermore, commercial LPS preparations, even when highly purified (≥97%), may contain trace amounts of other bacterial PAMPs such as peptidoglycan. Landmark studies in vertebrates demonstrated that immune responses initially attributed to LPS were later shown to involve PGN recognition pathways [40]. Therefore, while our results clearly demonstrate immune activation following endotoxin exposure, we interpret these findings as responses to bacterial PAMP stimulation rather than exclusively LPS-specific recognition. Future studies employing ultrapure LPS, enzymatic PGN degradation controls, or receptor-specific assays will help clarify the molecular basis of endotoxin recognition in Arbacia punctulata.

It is important to consider that elevated ammonia concentrations observed in the stressed tanks may themselves represent an additional physiological stressor. Ammonia is known to impair development, acid-base balance, and cellular homeostasis in marine invertebrates [18]. Therefore, some physiological alterations observed in our study, particularly changes in coelomic protein levels and GSI, may reflect cumulative effects of primary stressors (temperature and endotoxin exposure) combined with secondary ammonia stress. Future studies should incorporate controlled water exchanges or ammonia buffering systems to isolate direct effects of thermal and PAMP stressors from nitrogenous waste accumulation.

## 5. Conclusion

Our findings suggested that both increased temperature and LPS inoculation have harmful impacts on sea urchin, *Arbacia punctulata*. As coelomocytes and coelomic proteins are the main defense system for sea urchins, we observed that stressors (physical and chemical) caused abnormal physiological responses (increased or decreased in coelomocytes and coelomic protein), less gonad, and eventually made them immunosuppressed. Physical and chemical stressors can create stress for sea urchins both in natural environment and in echinoderm culture. To conserve the natural stock of the sea urchin population and to produce healthier sea urchins commercially we need to ensure stress-free environments. However, more research is still required in this area.

## Supporting information

S1 File Nahian Supporting Info Plos 1. (XLSX)
